# The Physical Impact of a Rare Lysosomal Storage Disorder: Scheie Syndrome in Primary Care

**DOI:** 10.51894/001c.144656

**Published:** 2025-09-30

**Authors:** Taylor Totterdale

**Affiliations:** 1 Department of Family Medicine McLaren Greater Lansing

## 130

### ABSTRACT

Scheie Syndrome, also known as Mucopolysaccharidosis I (MPS-I), comprises a multitude of symptoms that may arise when pathogenic variants of genes coding lysosomal enzyme alpha-L iduronidase lead to accumulation of glycosaminoglycan fragments within lysosomes. Symptoms may include stiff joints, corneal clouding requiring corneal transplant, and cardiac valvular disease. This case highlights the importance of recognizing MPS-I and its physical impact in the primary care setting, as it follows a 52-year-old male with tendon contractures, diastolic heart failure, aortic stenosis, mitral stenosis, lower extremity edema, hyperlipidemia, and glaucoma with bilateral corneal transplant. He presented to establish care with a local family medicine residency clinic. Though he followed with Rheumatology for once-weekly Laronidase infusions (AldurazymeⓇ) for MPS-I, at the time of presentation, he had missed eight contiguous infusions. Physical examination was significant for the use of a cane to walk as well as shortening with contracture of all distal phalanges and worsening ability to close his fingers into a fist. Ultimately, the patient was amenable to obtaining a physical therapy evaluation to help with muscle and gait training and contacting his rheumatologist to restart the Laronidase infusions. Follow-up will consist of continued strengthening of weakened muscle and management of joint dysfunction that developed through inadequate medical management. While Scheie syndrome is a rare presentation in the primary care setting, it is vital to recognize its detrimental effects and provide resources for regaining strength through physical therapy and Laronidase infusions.

